# Genomic discovery of the hypsin gene and biosynthetic pathways for terpenoids in *Hypsizygus marmoreus*

**DOI:** 10.1186/s12864-018-5159-y

**Published:** 2018-11-01

**Authors:** Byoungnam Min, Seunghwan Kim, Youn-Lee Oh, Won-Sik Kong, Hongjae Park, Heejung Cho, Kab-Yeul Jang, Jeong-Gu Kim, In-Geol Choi

**Affiliations:** 1 Department of Biotechnology, College of Life Sciences and Biotechnology, Korea University, 145 Anam-ro, Seongbuk-Gu, Seoul, 02841 Korea; 2 Genomics Division, National Institute of Agricultural Sciences, Rural Development Administration (RDA), Jeonju, 54874 Korea; 3 Mushroom Division, National Institute of Horticultural and Herbal Science (NHHS), Rural Development Administration (RDA), Eumseong, 27709 Korea

**Keywords:** *Hypsizygus marmoreus*, Beech mushroom, Fungal genome, Hypsin, Marmorin, Hypsiziprenol A9, Secondary metabolism

## Abstract

**Background:**

*Hypsizygus marmoreus* (Beech mushroom) is a popular ingredient in Asian cuisine. The medicinal effects of its bioactive compounds such as hypsin and hypsiziprenol have been reported, but the genetic basis or biosynthesis of these components is unknown.

**Results:**

In this study, we sequenced a reference strain of *H. marmoreus* (Haemi 51,987–8). We evaluated various assembly strategies, and as a result the Allpaths and PBJelly produced the best assembly. The resulting genome was 42.7 Mbp in length and annotated with 16,627 gene models. A putative gene (Hypma_04324) encoding the antifungal and antiproliferative hypsin protein with 75% sequence identity with the previously known N-terminal sequence was identified. Carbohydrate active enzyme analysis displayed the typical feature of white-rot fungi where auxiliary activity and carbohydrate-binding modules were enriched. The genome annotation revealed four terpene synthase genes responsible for terpenoid biosynthesis. From the gene tree analysis, we identified that terpene synthase genes can be classified into six clades. Four terpene synthase genes of *H. marmoreus* belonged to four different groups that implies they may be involved in the synthesis of different structures of terpenes. A terpene synthase gene cluster was well-conserved in Agaricomycetes genomes, which contained known biosynthesis and regulatory genes.

**Conclusions:**

Genome sequence analysis of this mushroom led to the discovery of the hypsin gene. Comparative genome analysis revealed the conserved gene cluster for terpenoid biosynthesis in the genome. These discoveries will further our understanding of the biosynthesis of medicinal bioactive molecules in this edible mushroom.

**Electronic supplementary material:**

The online version of this article (10.1186/s12864-018-5159-y) contains supplementary material, which is available to authorized users.

## Background

**Fig. 1:**
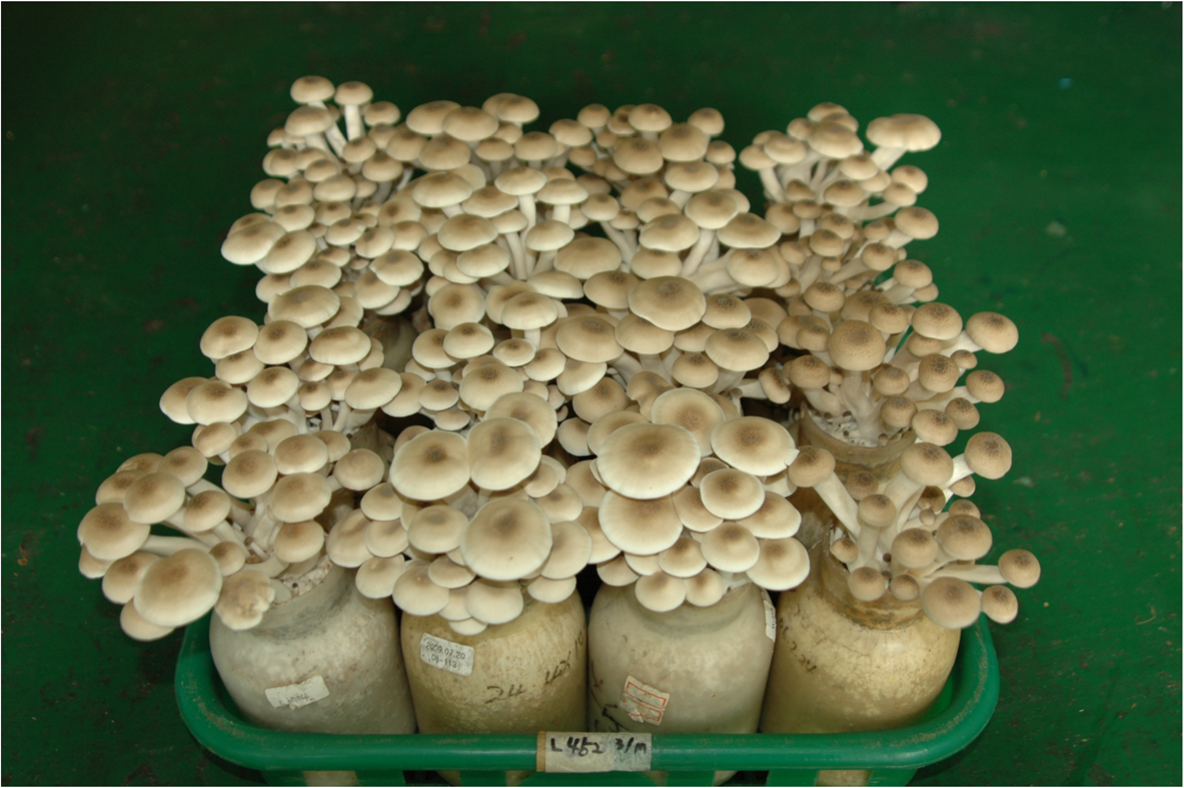
Fruiting bodies of *Hypsizygus marmoreus*

*Hypsizygus marmoreus* is an edible mushroom with various medicinal effects, including antitumor, antibacterial, and antifungal properties [1–4] (Fig. 1). Several bioactive molecules have been reported to underlie the medicinal effects of *H. marmoreus*; in particular, the terpenoid compound hypsiziprenol A9 inhibits cell cycle progression in HepG2 cells, a human liver cancer cell line [2]. The thermostable ribosome-inactivating protein hypsin, which can be extracted from the fruiting body of the mushroom, has antifungal and antiproliferative properties [5]. Another ribosome-inactivating protein, marmorin, has antiproliferative and HIV-1 reverse transcriptase inhibitory activities [6]. Despite the popularity of *H. marmoreus* as a gastronomic and medicinal resource, the genetic basis or biosynthetic pathways of these active compounds are unknown. Using genome sequencing, we aim to understand the mushroom’s bioactivity at the genomic level.

Various terpenoid compounds from different mushrooms have been reported to have medicinal effects [7]. Genome sequencing methods have been used to elucidate the biosynthetic pathways of terpenoid compounds by identifying terpene synthase genes. For example, potential terpene synthase genes in *Coprinus cinereus* [8], *Omphalotus olearius* [9], and *Stereum hirsutum* [10] have been mined via genome sequencing, and their biochemical activities have been studied. In particular, coexpression of *cop6* and the two P450 monooxygenase genes of *C. cinereus* has been reported to produce the antimicrobial compound lagopodin [8]. Thus, it is important to combine molecular, genetic, and biochemical techniques within the genomic context to understand the biosynthesis of natural bioactive compounds. Both biochemical compounds and many proteins, such as lectins, fungal immunomodulatory proteins, ribosome-inactivating proteins, ribonucleases, and laccases, have been suggested as candidates for medicinally active components in mushrooms [11]. Ribosome-inactivating proteins inhibit protein synthesis by modifying ribosomal RNA. This results in HIV-1 reverse transcriptase inhibition as well as antifungal, anticancer, and antiproliferative activities [11]. Plants are the primary sources of these ribosome-inactivating proteins [3] and some mushrooms. Examples of ribosome-inactivating proteins expressed in mushrooms include velutin (*Flammulina velutipes*) [12], flammulin (*F. velutipes*) [13], and lyophyllin (*Lyophyllum shimeji*) [14]. Genes encoding these proteins have not yet been explored despite the availability of genome sequences for these species [15, 16].

In this study, we reported the fully annotated genome of *H. marmoreus* and elucidated the genetic basis of the biosynthesis of bioactive molecules reported in this mushroom. We obtained a high-quality genome assembled from three different sequencing libraries using multiple genome assembly strategies. Sequential and functional comparisons enabled us to identify the hypsin gene in the genome. Orthologous gene analysis identified putative genes responsible for biosynthesizing hypsiziprenol A9.

## Results

### Genome assembly using various strategies

We constructed and sequenced three genomic DNA sequencing libraries: paired-end and mate-pair Illumina libraries and a PacBio library (Table 1). To obtain a high-quality genome assembly, we applied five assembly strategies to the assembly procedure: (i) Allpaths, (ii) Allpaths+PBJelly, (iii) Allpaths+SSPACE-longread, (iv) Falcon, and (v) SPAdes. Allpaths assembled the two Illumina libraries, and PBJelly [17] and SSPACE-longread [18] individually improved the assembly with PacBio reads. SPAdes [19] used all three libraries for a hybrid assembly. Because we had over 200× physical coverage of PacBio reads, we also sequentially used Falcon [20], FinisherSC [21], and Quiver [22] for PacBio-only assembly. The results of the five assembly strategies are summarized in Table 2. From the assembly assessment, we selected the Allpaths+PBJelly assembly for further analyses (See Discussion).

**Table 1:** Sequencing data summary

Type	Library	Insert size	Average read size	Number of total reads
Genome	Illumina paired-end	400 bp	300 bp	14,888,962 × 2
	Illumina mate-pair	5000 bp	100 bp	157,055,636 × 2
	PacBio	–	7980 bp	1,125,617 (6 cells)
Transcriptome	Illumina paired-end 1	–	100 bp	25,218,416 × 2
	Illumina paired-end 2	–	100 bp	89,796,090 × 2

The two transcriptome libraries are technical replicates of the same sample

**Table 2:** Preliminary assemblies using five assembly strategies

Metrics	Allpaths	Allpaths+PBJelly	Allpaths+SSPACE-longread	SPAdes	Falcon
Libraries	Paired-end Mate-pair	Paired-end Mate-pair PacBio	Paired-endMate-pair PacBio	Paired-end Mate-pair PacBio	PacBio
Number of scaffolds	340	235	150	199	59
Number of contigs	1000	278	1018	261	59
Assembly size (Mbp)	41.6	42.7	42.3	42.1	42.2
N50 value (scaffolds)	628.3 kbp	764.8 kbp	947.1 kbp	1.1 Mbp	1.6 Mbp
N50 value (contigs)	152.4 kbp	621.3 kbp	149.5 kbp	766.3 kbp	1.6 Mbp
Number of scaffolds > 1 Mbp (scaffold sizes sum)	5 (8.4 Mbp)	7 (12.9 Mbp)	11 (18.6 Mbp)	12 (22.8 Mbp)	15 (29.2 Mbp)
Complete BUSCOs	1298	1304	1300	1301	1298
Fragmented BUSCOs	24	20	22	21	22
Missing BUSCOs	13	11	13	13	15
CGAL value	−3.61e + 09	−3.52e + 09	−3.90e + 09	−1.12e + 09	−2.83e + 09

The final assembly had a size of 42,710,661 bp including 235 scaffolds/278 contigs with 287.3× sequence coverage. The GC percentage was 49.64%. We estimated the genome size as 43.0 Mbp using the k-mer frequency calculation of Illumina paired-end reads (Additional file 1**:** Figure S1). We confirmed that the assembly was a haploid genome rather than a diploid genome from the single peak in the k-mer frequency plot (Additional file 1: Figure S1). Many mushrooms were in the dikaryon stage, which introduced diploidy into their assembly. This impedes interpretation of the genome because it is difficult to differentiate between duplication and diploidy. We analyzed the ploidy of the assembly by drawing a read coverage histogram and confirmed that the genome was monokaryotic (Additional file 1: Figure S2). This was consistent with the results of the k-mer frequency estimation. There was no obvious mitochondrial or contaminated sequence in the assembly (Additional file 1: Figure S3).

### Repeat elements

To avoid spurious gene prediction due to repeats, we identified a total of 2,482,387 bp (5.81%) interspersed repeat regions. This included 28 long interspersed nuclear element (7629 bp), 867 long terminal repeat elements (887,560 bp), 586 DNA elements (378,418 bp), and 2044 unclassified elements (1,208,780 bp). We masked these regions for gene prediction.

### Genome annotation

Using the FunGAP pipeline [23], we predicted 16,627 protein-coding genes with an average size of 1586.1 nt. Of these protein-coding genes, 14,179 genes (85.3%) were supported by assembled transcripts, and this included 10,522 (63.3%) highly supported genes (> 90% coverage). Genome completeness was calculated using BUSCO v3.0 at the gene level. Only 5 of 1335 single-copy entries were missing, indicating > 99% genome completeness. The quality of the gene prediction was evaluated by comparing the predictions of three programs inside the FunGAP pipeline: Augustus 3.2.1 [24], Braker 1.8 [25], and Maker 2.31.8 [26] (Additional file 2: Table S1). Gene prediction results are summarized in Table 3.

**Table 3:** Gene prediction summary

Attributes	Values
Total protein-coding genes	16,627
Transcript length (average/median)	1586.1/1316
CDS length (average/median)	1275.5/1038
Protein length (average/median)	425.2/346
Exon length (average/median)	221.8/129
Intron length (average/median)	65.4/55
Spliced genes	14,685 (88.32%)
Gene density (genes/Mb)	389.29
Coding regions	49.65%
Number of introns	79,007
Number of introns per gene (med)	4
Number of exons	95,634
Number of exons per gene (med)	4

Approximately half of the predicted genes were functionally annotated; in total, 7786 genes (46.8%) were annotated using Pfam domains, and 7447 genes (44.8%) were annotated using SwissProt. The dominant functions included WD, F-box, protein kinase, cytochrome P450, and major facilitator superfamily domains, similarly as observed in other mushroom genomes [27, 28]. The genome contained 1793 genes encoding secreted proteins. We identified 1262 noncoding RNA elements containing 171 tRNAs, including 9 selenocysteine tRNAs, 191 small nucleolar RNAs (snoRNAs) from 127 different families, and 224 microRNAs from 90 different families.

### Phylogenetic location in Agaricomycetes

**Fig. 2:**
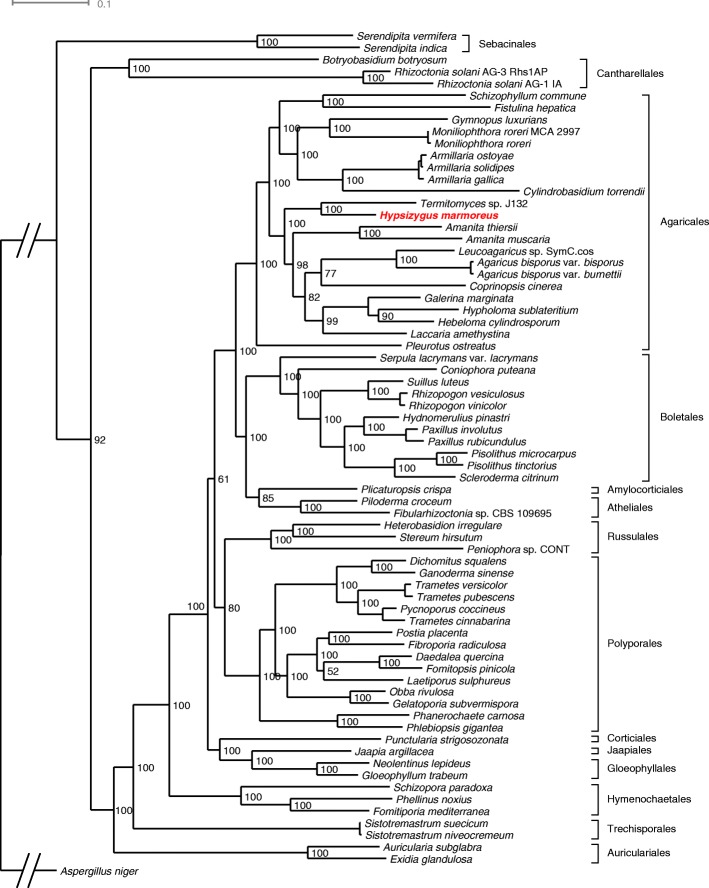
Phylogenetic location of *Hypsizygus marmoreus* among Agaricomycetes. Phylogeny was inferred from 102 concatenated single-copy orthologs using RAxML 8.1.3. Bootstrapping-based branch support and a scale bar representing the mean number of amino acid substitutions per site are shown

In March 2017, 85 Agaricomycetes genome assemblies with predicted genes were present in the NCBI database. We excluded 15 genomes with low BUSCO completeness (< 95%); thus, we compared our assembled *H. marmoreus* genome with 69 reference genomes (Additional file 2: Table S2). In the resulting genome tree, *H. marmoreus* clustered with other Agaricales species, with *Termitomyces* identified as the closest relative (Fig. 2). Agaricomycetes species had genome sizes of 25–119 Mbp with 9262–32,854 genes. The genome size and gene number of *H. marmoreus* were close to the average of these distributions (Additional file 1: Figure S4).

### Carbohydrate active enzymes (CAZymes)

**Fig. 3:**
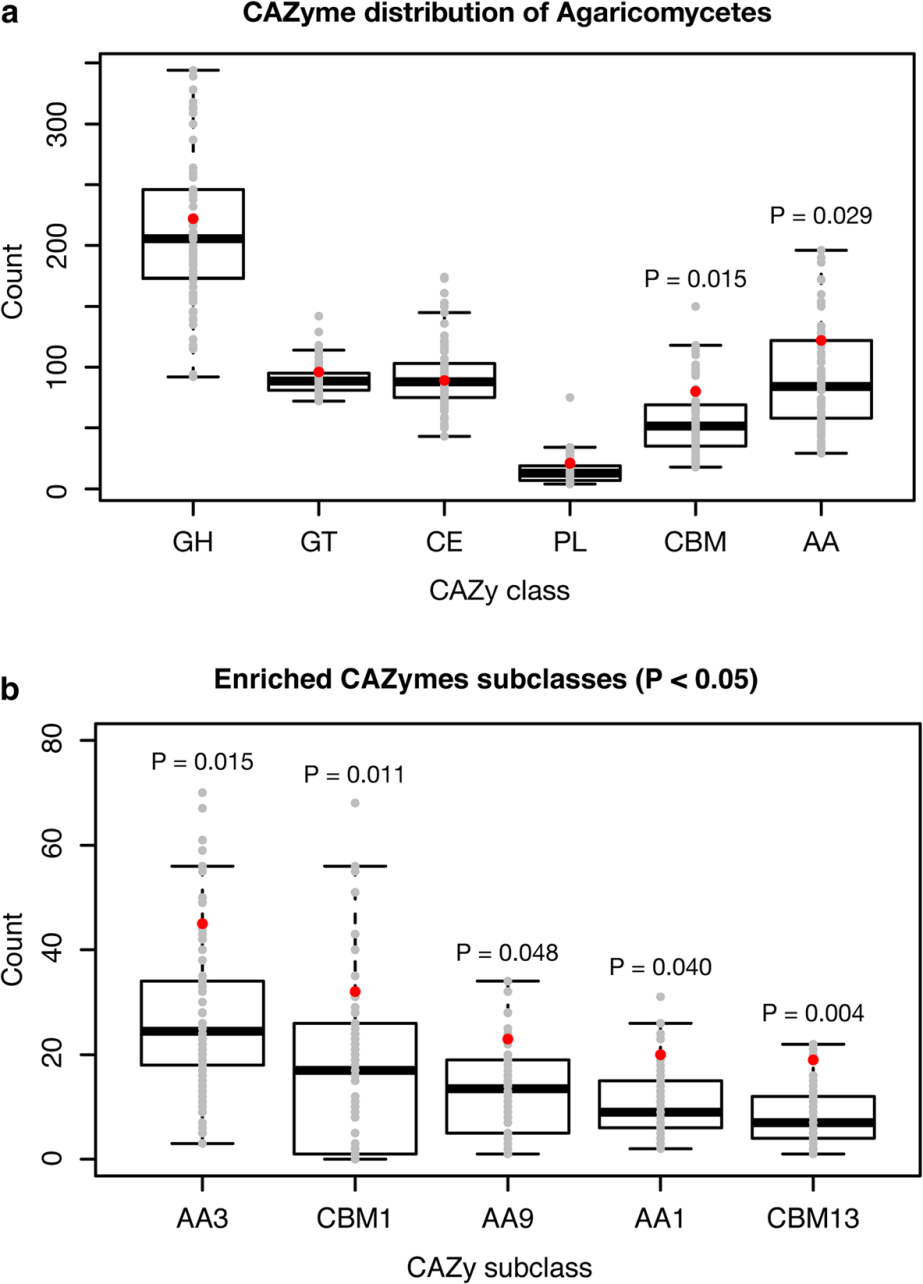
Carbohydrate active enzymes (CAZymes) in Agaricomycetes genomes. **a** Distribution of six CAZyme classes. The *P* values were calculated using the Scipy (https://www.scipy.org) stats.fisher_exact function, which performs Fisher’s exact test. Only significant P values (*P* < 0.05) are indicated. Red points indicate the *Hypsizygus marmoreus* genome. **b** Enriched CAZyme subclasses in the *H. marmoreus* genome (P < 0.05). Significantly depleted CAZyme subclasses were not identified

The *H. marmoreus* genome contained a total of 630 CAZyme modules within 590 genes, including 222 glycoside hydrolases (GHs), 96 glycosyltransferases, 89 carbohydrate esterases, 21 polysaccharide lyases, 80 carbohydrate-binding modules (CBMs), and 122 auxiliary activity (AA) modules (Fig. 3a). Compared with other Agaricomycetes genomes, the *H. marmoreus* genome was enriched in AA and CBM modules (*P* < 0.05). The reference genomes had median values of 91 AA and 55 CBM modules. In detail, five subclasses were particularly enriched in *H. marmoreus*: AA1, AA3, AA9, CBM1, and CBM13 (P < 0.05, Fig. 3b). AA1, AA3, and AA9 encode multicopper oxidases, glucose–methanol–choline oxidoreductases, and copper-dependent lytic polysaccharide monooxygenases, respectively. These genes are well-known lignocellulose-degrading enzymes [29]. As *H. marmoreus* is known as a white-rot fungus [30], these enriched CAZyme families are congruent with the representative feature of white-rot fungal genomes [31]. The cellulose-binding CBM1 module is generally enriched in white-rot fungal genomes, whereas brown-rot fungal genomes have none or a few of these modules [31]. We identified 25 CBM1 modules in the genome. These modules were found with various CAZyme modules including GH6, GH7, and AA9 in various genes, which may lead to synergetic degradation. Whereas CBM13 modules related to the ricin-type beta-trefoil lectin domain (Pfam: PF00652) are found as part of many carbohydrate-binding proteins [32–34], none of these domains were accompanied by other CAZyme modules. This suggests that CBM13-containing proteins in this genome are not involved in carbohydrate degradation processes. Instead, all these proteins were extracellular proteins. Further experimental verification is needed to reveal their biological and molecular functions. In summary, the *H. marmoreus* CAZyme profile revealed the features of white-rot fungi with enriched lignocellulose-degrading enzymes.

### Candidate hypsin and marmorin genes

**Fig. 4:**
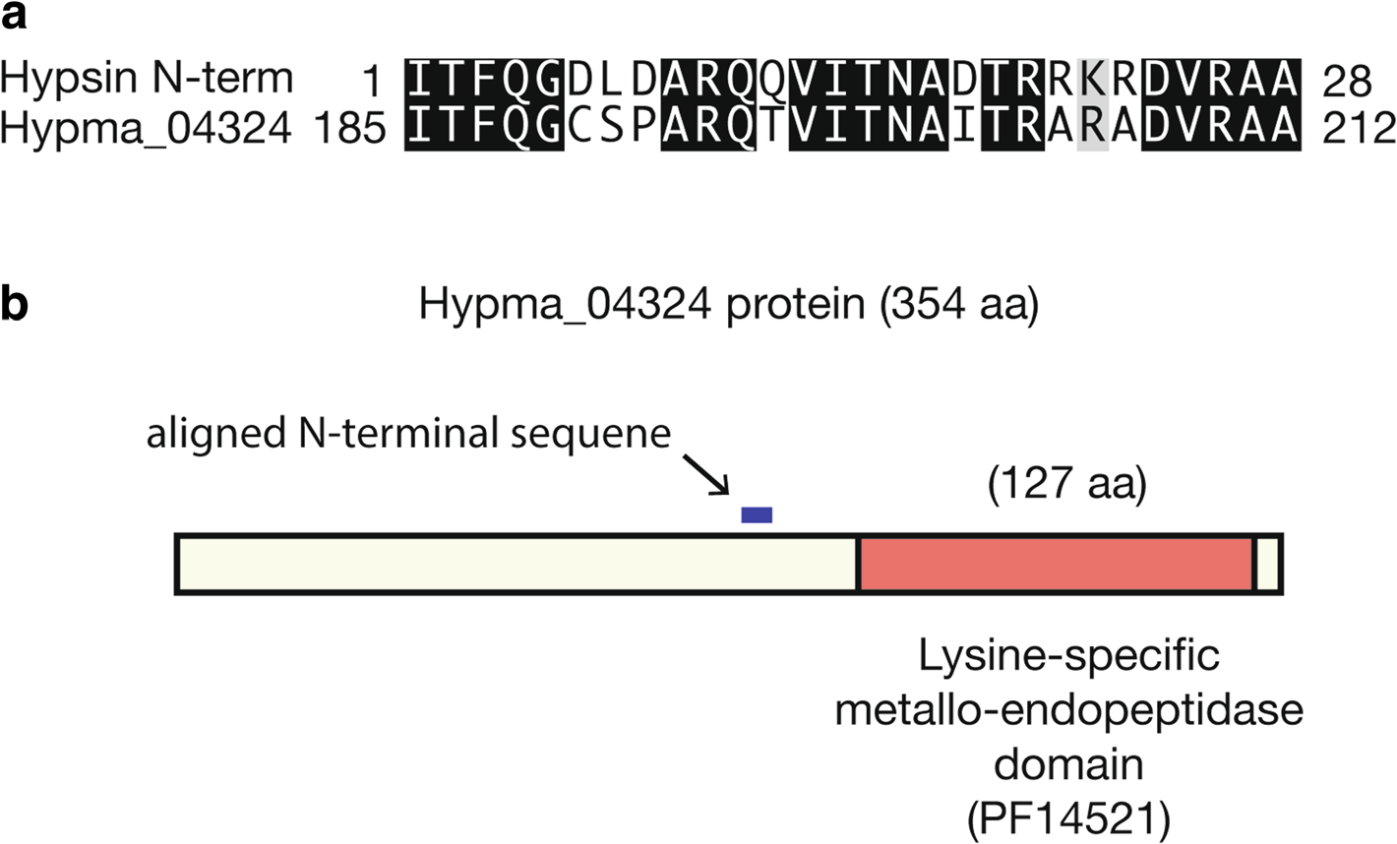
A candidate hypsin gene obtained using sequence alignment. **a** Alignment of the experimentally determined N-terminal sequence of hypsin and BLAST-searched Hypma_04324. **b** Schematic representation of the Hypma_04324

Hypsin and marmorin are two major bioactive proteins previously reported as ribosome-inactivating proteins with antiproliferative activities against tumor cells [5, 6]. The N-terminal sequences of both proteins are “ITFQGDLDARQQVITNADTRRKRDVRAAVR” (28 amino acids) for hypsin and “AEGTLLGSRATCESGNSMY” (19 amino acids) for marmorin. The N-terminal sequence of hypsin was similar to those of plant ribosome-inactivating proteins, such as alpha-momorcharin [35] and trichosanthin [36]. The molecular weights were 20 and 9.5 kDa for hypsin and marmorin, respectively. We searched the entire genome for these N-terminal sequences and identified a potential hypsin gene (Hypma_04324) with 71% identity (20/28 matches) and 75% positive matches (21/28) (Fig. 4).

### Secondary metabolism genes

**Fig. 5:**
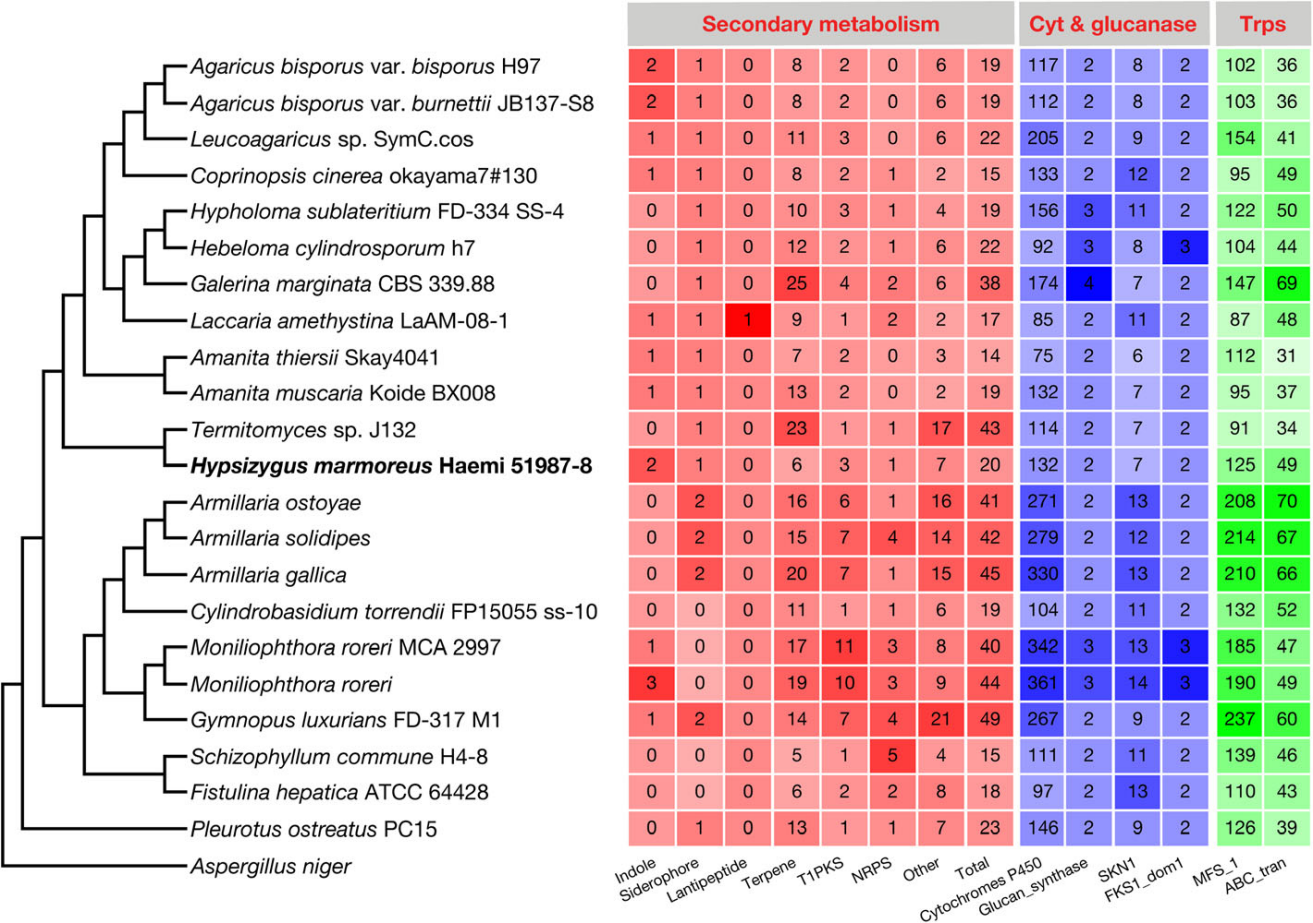
Secondary metabolism genes of 22 Agaricales species. Detailed methods for building the genome tree and predicting secondary metabolism genes are described in the Methods section. The genomes are listed in Additional file 2: Table S2. *Aspergillus niger* was used as an outgroup

The *H. marmoreus* genome contained 20 secondary metabolism gene clusters, including six terpene/phytoene synthases, three type-I polyketide synthases, one siderophore synthase, one nonribosomal peptide synthase, two indole synthases, and seven unknown clusters. Agaricales genomes contained an average of 27 gene clusters (range, 14–49) (Fig. 5). Type-III polyketide synthases, which have been functionally characterized in several ascomycetes [37], were lacking in all Agaricales genomes. However, the *Phanerochaete carnosa* (Polyporales) and *Exidia glandulosa* (Auriculariales) genomes contained one copy each (Additional file 2: Table S3).

Cytochrome P450 has an important role in modifying backbone secondary metabolites, such as lanosterol [38]. The *H. marmoreus* genome contained 132 cytochrome P450 domains. The two *Moniliophthora* genomes contained the largest numbers of this domain (342 and 361 domains, respectively, Fig. 5). Glucan synthases produce various glucan compounds with bioactive properties. All Agaricales genomes contained 2–4 glucan synthase genes, with *H. marmoreus* containing two. Major facilitator superfamily and ABC transporters are important in transporting secondary metabolites [39]. We found 125 and 49 major facilitator superfamily and ABC transporter genes, respectively, in the *H. marmoreus* genome.

### Sesquiterpene synthases

**Fig. 6:**
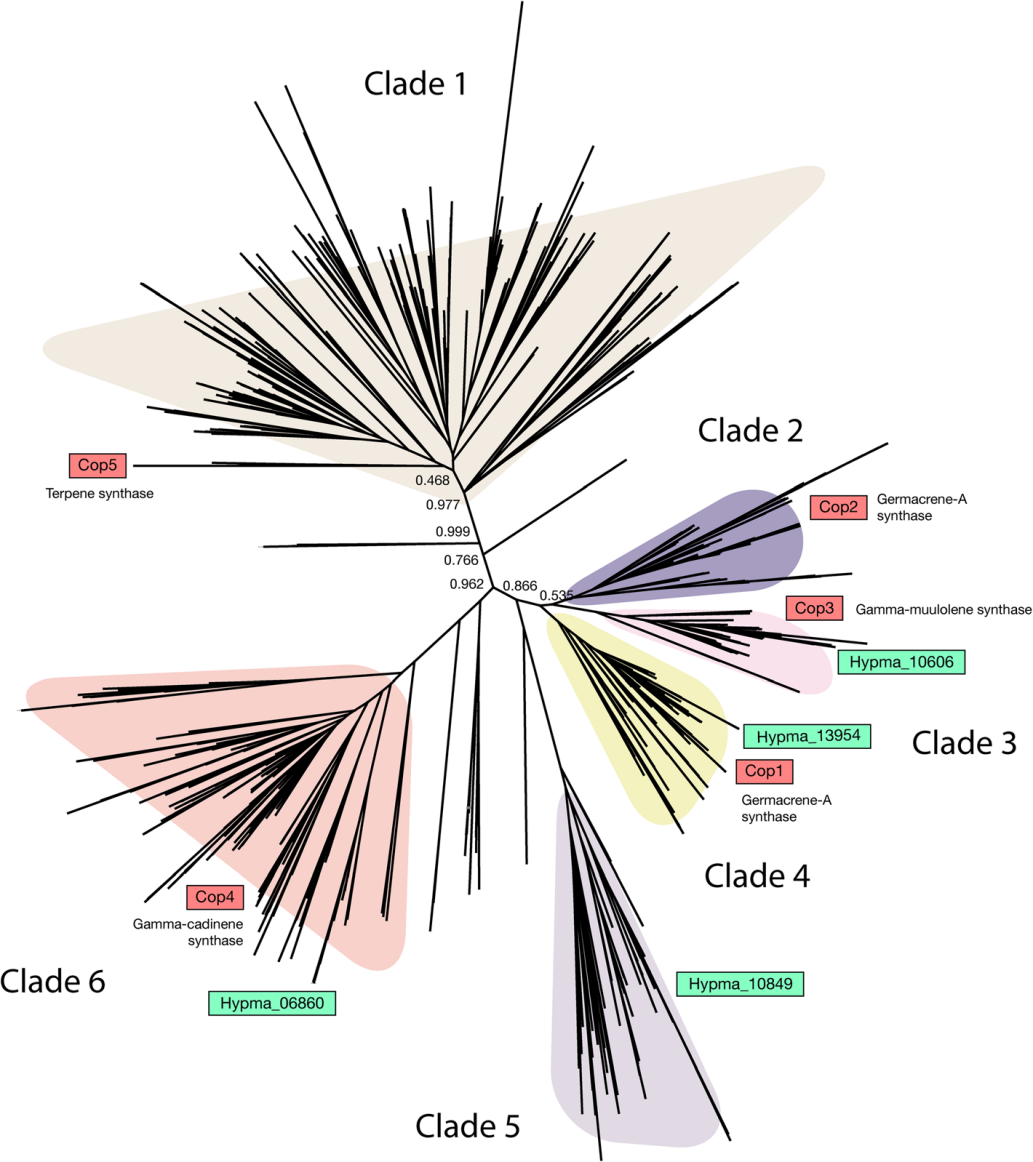
A gene tree of terpene synthases. We used 759 putative terpene synthase genes from an orthologous group containing known terpene synthase genes

Various terpenoid compounds are produced by biosynthetic clusters. *H. marmoreus* produces the terpene compound hypsiziprenol A9, which has antitumor properties [2]. To elucidate the conserved and diverged structures of terpenoid gene clusters, we obtained 759 terpene synthase genes from the 70 Agaricomycetes genomes. The Agaricomycetes genomes contained 1–25 terpene synthase genes, including four genes in the *H. marmoreus* genome (Additional file 1: Figure S5). From the gene tree, we identified six groups of terpene synthase genes, as reported previously [8, 10] (Fig. 6 and Additional file 1: Figure S6). The orthologs of three well-characterized *C. cinereus* terpene synthases, Cop1, Cop3, and Cop4, were identified in the *H. marmoreus* genome.

**Fig. 7:**
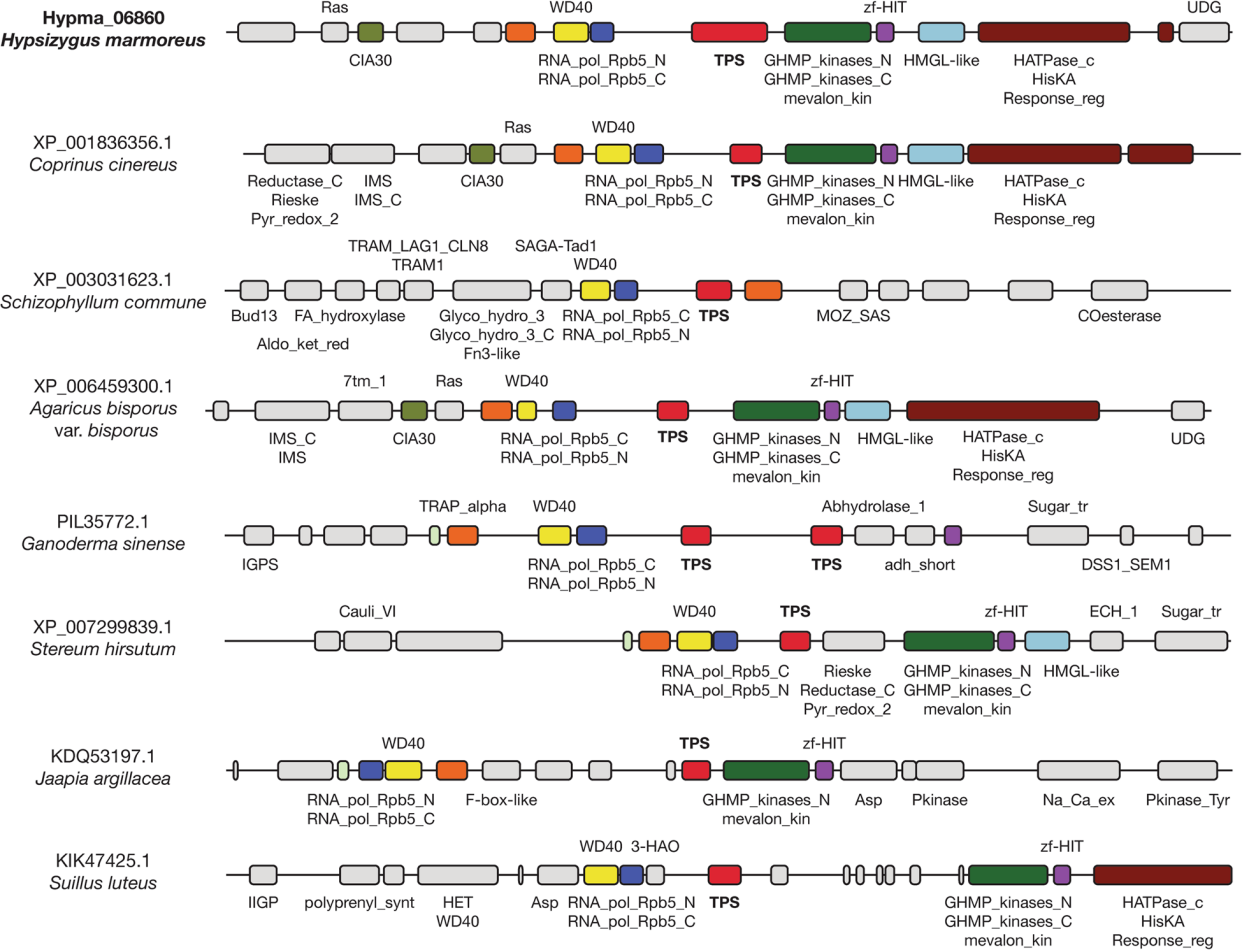
Arrangement of conserved neighboring genes surrounding terpene synthase among Agaricomycetes species (clade 6). The figure displays 40-kbp regions encompassing terpene synthases in which each gene is labeled with Pfam annotation when available. Orthologous genes are marked with the same color

Various functional genes are clustered with terpene synthase genes, including terpene-modifying enzymes, regulatory proteins, and transporters (Fig. 7 and Additional file 1: Figures S7–S9). In particular, clade 6 terpene synthase genes had well-conserved gene clusters across all Agaricomycetes orders including Agaricales, Boletales, Polyporales, Russulales, and Jaapiales. This cluster contained galacto-kinase, homoserine-kinase, mevalonate-kinase, phosphomevalonate-kinase (GHMP kinase, Pfam: PF00288, PF08544), HMGL-like domain (pyruvate carboxylase, Pfam: PF00682), HIT zinc finger (Pfam: PF04438), and response regulator receiver domain (Pfam: PF00072) (Fig. 7). GHMP kinase and HMGL-like domains are directly related to the biosynthesis of terpenoids [40, 41]. HIT zinc finger and response regulator receiver domains are related to gene regulation [42, 43]. These well-conserved gene clusters suggest the putative resemblance of their product structure and their regulation. Conversely, clade 5 lacked a conserved gene cluster. In other gene clusters, transporter (major facilitator superfamily), heat shock protein activator, helicase, and F-box-like domains were frequently identified. Their exact molecular functions and associations with terpene synthesis remain to be elucidated by in vitro/in vivo experiments. The terpene synthases and their adjacent genes displayed transcriptional activity in hyphae (Additional file 1: Figure S10). This suggests that they are coregulated, although further investigation is needed to reveal whether they are related to the biosynthesis of a terpenoid.

## Discussion

### Genome assembly assessment

The results of the five preliminary assemblies were evaluated using two approaches: BUSCO v3.0 completeness calculation [44] and CGAL assembly likelihood calculation [45]. Falcon assembly displayed the lowest number of scaffolds and the highest N50 value. However, it was the most incomplete assembly, as it lacked the highest number of BUSCO entries. This implies that this assembly misses some genomic regions that could contain protein-coding genes. Scaffolding with SSPACE-longread on Allpaths assembly decreased the number of scaffolds by nearly half, but the actual number of contigs increased. SPAdes using all three libraries generated comparable assemblies as the other libraries with the lowest CGAL value. In terms of genome completeness inferred by BUSCO, Allpaths+PBJelly displayed the best quality among the five candidates with the largest assembly size (42.7 Mbp). For further investigation, we predicted protein-coding genes in the Allpaths+PBJelly and Falcon assemblies. There were 571 more genes predicted in the Allpaths+PBJelly assembly than in the Falcon assembly (Additional file 2: Table S4). In particular, there were nine Pfam domains that were not found in the Falcon assembly gene prediction, which could affect downstream functional analyses. Sequence alignment revealed 1.27 Mbp and 782 kbp of unique regions in the Allpaths+PBJelly and Falcon assemblies against each other, respectively. One unique region in the Allpaths+PBJelly assembly included a missing BUSCO entry. Although the Allpaths+PBJelly assembly was rather fragmented compared to the assemblies from the other methods, the Allpaths+PBJelly assembly contained more genomic regions, implying more completeness for functional and comparative analyses. Thus, we selected the Allpaths+PBJelly assembly as the final assembly for the subsequent analyses.

### Putative hypsin gene

The putative hypsin gene had 71% identity to reported hypsin N-terminal sequence. The discrepancy may be due to their regional origins, as the reported *H. marmoreus* was isolated from China whereas our sample was obtained from Korea. The matched region with the known N-terminal sequence was located starting at amino acid 185. The calculated weight of the truncated protein from the matched region was estimated as 18.2 kDa, which is similar to the reported molecular weight (20 kDa) of hypsin. As the reported hypsin was purified from cell extracts, the hypsin gene identified in this genome might be post-translationally processed to an active form. Interestingly, the hypsin gene contained a lysine-specific metallo-endopeptidase (Pfam: PF14521, peptidase M35 family) domain at amino acids 223–349 but displayed no significant sequence similarity with alpha-momorcharin or trichosanthin. This protein was predicted to have a signal peptide for secretion, similarly as other peptidase M35 family proteins. The *H. marmoreus* genome contained four copies of this protein, which is common in other Agaricomycetes. Specifically, 39 of 70 genomes contained at least one ortholog, and the *Rhizoctonia solani* genome had 38 copies. The exact molecular function and medicinal effects of this candidate hypsin remain to be elucidated. We were unable to find a candidate gene for another known bioactive protein, marmorin, in the current gene prediction and genome assembly.

## Conclusions

We constructed a high-quality genome assembly and annotation for the genes and gene clusters of medicinal compounds. Thus, this study serves as a primary case study for combining experimental results and the genomics of mushrooms containing highly valuable bioactive compounds.

## Methods

### Library preparation and sequencing

The mycelium of *H. marmoreus* Haemi 51,987–8 (Korean Collection for Type Cultures No. 46454, http://kctc.kribb.re.kr/En/Kctc.aspx) was cultured in 65% potato dextrose broth (BD Difco™, Franklin Lakes, NJ, USA) under shaking at 24 °C for 3–7 days. The genomic DNA of the monokaryotic strain was extracted from mycelia using a DNeasy Plant Mini Kit (Qiagen, Valencia, CA, USA). Three libraries were generated for genome assembly: paired-end and mate-pair Illumina libraries and a PacBio library. In total, 40 and 9 Gbp of data were generated using Illumina and PacBio, respectively. RNA molecules were extracted from mycelia using an RNeasy Plant Mini Kit (Qiagen). Two mRNA sequencing libraries were generated for gene prediction (23 Gbp). Illumina reads were trimmed and filtered by base quality and read length using HTQC 1.92.3 [46], and PacBio reads were filtered by read length and read quality using SMRT analysis 2.3.0 *RS_Subreads* protocol (https://www.pacb.com/products-and-services/analytical-software/smrt-analysis/). The sequenced data are summarized in Table 1.

### Genome size estimation

The genome size of *H. marmoreus* was estimated using k-mer frequency. The paired-end Illumina library was used to draw k-mer frequency plots, in which 17 and 19 k-mers, respectively, were set. JellyFish 2.2.4 [47] was used to calculate the frequencies.

### Genome assembly

Using three different genomic DNA libraries (Illumina paired-end, Illumina mate-pair, and PacBio), we built five candidate assemblies using the following strategies: (i) Allpaths, (ii) Allpaths+PBJelly, (iii) Allpaths+SSPACE-longread, (iv) Falcon, and (v) SPAdes. First, Illumina paired-end and mate-pair libraries were used to run Allpaths [48] with PLOIDY = 1. This assembly was improved by running PBJelly 15.8.24 [17] and SSPACE-longread v1.1 [18], which both require filtered subreads (filtered PacBio reads) as an input. Falcon [20] was run using only PacBio reads with a cutoff length of 8000 bp. FinisherSC [21] and Quiver [22] were run to improve the Falcon assembly. We also used SPAdes v3.10.1 [19] for a hybrid assembly using all three libraries with the *--careful* option. The five assembly candidates were evaluated using BUSCO 3.0.2 [44] with *basidiomycota_odb9* lineage data and CGAL 0.9.6 [45].

### Post-process of the assembly

We checked whether the assembly contained mitochondrial or contaminated sequences. In a fungal genome assembly, the mitochondrial genome generally has a much higher sequence depth and a lower GC content than the nuclear genome [49]. We ran BLASTn with the assembly against the NCBI mitochondrial genome database (ftp://ftp.ncbi.nlm.nih.gov/blast/db/FASTA/mito.nt.gz). Hit scaffolds were investigated for their read coverages and GC contents if they were outliers relative to other scaffolds. Sequence contamination was checked using Blobology 2015-12-16 [50].

### Genome annotation

We first detected repeat regions to mask before gene prediction using RepeatModeler and RepeatMasker (http://www.repeatmasker.org). Protein-encoding genes in the assembly were predicted using the FunGAP pipeline. mRNA sequences were sampled into 58.8 million reads (5.83 billon bases) to decrease the computing time. mRNA reads were mapped into the genome using Hisat 2.0.2 [51]. The mapped reads were assembled using Trinity 2.2.0 [52]. *Laccaria bicolor* was set as the Augustus [24] species model. Noncoding RNA elements such as tRNAs, snoRNAs, and microRNAs were annotated by scanning Rfam database release 12.1 [53] using Infernal 1.1.1 [54].

### Reference genomes for comparative analysis

We downloaded the protein sequences of 69 Agaricomycetes species, including *H. marmoreus*, in FASTA format from the NCBI database. We ran OrthoFinder 1.0.6 [55] to obtain orthologous genes from the genomes and selected 102 single-copy orthologs to build a species tree. The program was run with *Aspergillus niger* protein sequences comprising an outgroup (GenBank accession: GCF_000002855.3). RAxML 7.3.0 [56] was used to build the tree with “*-f a -x 12345 -p 12345 -# 100 -m PROTGAMMAWAG*” options. The tree was visualized using Dendroscope 3.5.9 [57].

### CAZyme analysis

CAZymes in the *H. marmoreus* genome and the 69 reference genomes were identified by combining dbCAN, BLASTp, and Pfam domain predictions. The dbCAN 5.0 database [58] was searched using *hmmscan* [59] with default options, and the result was parsed using a script (http://cys.bios.niu.edu/dbCAN/download/hmmscan-parser.sh). BLASTp was run against CAZyme protein sequences download from dbCAN (http://cys.bios.niu.edu/dbCAN/download/CAZyDB.03172015.fa) with an E-value cutoff of 1e − 10. For Pfam domain prediction, we ran InterProScan 5.25–64.0 [60] against the Pfam 31.0 database and extracted CAZyme domain-containing proteins. *Pfam-A.full* data was used to obtain Pfam domains associated with CAZymes. We annotated CAZymes when they were predicted identically by more than two methods and added Pfam and dbCAN-only predicted CAZymes after manual curation. Enriched or depleted CAZymes were estimated using Fisher’s exact test from the Python Scipy package (https://www.scipy.org/).

### Search for hypsin and marmorin

The N-terminal sequences of hypsin and marmorin, namely “ITFQGDLDARQQVITNADTRRKRDVRAA” and “AEGTLLGSRATCESGNSMY,” respectively, were retrieved from previous publications [5, 6]. BLASTp was used against all protein sequences. We also ran tBLASTn against the assembled transcripts and genome assembly for unannotated genes.

### Predicting secondary metabolism genes

AntiSMASH 4.0.1 [61] was used to predict secondary metabolism genes in the genomes. The annotation of secondary metabolism genes was based on Pfam notation as follows: cytochrome P450, PF00067; glucan synthase, PF02364, PF03935, and PF14288; and transporters, PF07690 and PF00005. To obtain the terpene synthase genes of 70 Agaricomycetes genomes, we selected an ortholog group in which known terpene synthase genes (Cop1–5 genes) are included. The ortholog groups were estimated using OrthoFinder 1.0.6 [55]. The group contained 759 gene members. Seventeen incomplete genes (not starting with methionine) were excluded. The resulting 742 genes were used to build a gene tree using Mafft 7.273 [62] and FastTree 2.1.3 [63] for sequence alignment and tree building, respectively.

## Additional files

Additional file 1: **Figure S1.** K-mer frequency of genomic reads. **Figure S2.** Sequence coverage histogram. **Figure S3**. Read coverage and GC content plot. **Figure S4.** Genome sizes and gene numbers of Agaricomycetes. **Figure S5.** Terpene synthase genes of 70 Agraicomycetes. **Figure S6.** The gene tree of terpene synthase genes of ten Agraicomycetes. **Figure S7.** Arrangement of conserved neighboring genes surrounding terpene synthase among Agaricomycetes (clade 3)*.* **Figure S8.** Arrangement of conserved neighboring genes surrounding terpene synthase among Agaricomycetes (clade 4)*.* **Figure S9.** Arrangement of conserved neighboring genes surrounding terpene synthase among Agaricomycetes (clade 5)*.* **Figure S10.** Transcriptional expression of terpene synthase genes and their neighboring genes. (PDF 2772 kb)

Additional file 2: **Table S1.** Gene predictions of various programs. **Table S2.** Reference genomes for comparative analysis. **Table S3.** Secondary metabolism genes in the 70 Agaricomycetes genomes. **Table S4.** Gene predictions of genome assemblies generated by different methods. (XLSX 29 kb)

## Abbreviations

AA: Auxiliary activity
CAZyme: Carbohydrate active enzyme
CBM: Carbohydrate-binding module
CE: Carbohydrate esterase
GH: Glycoside hydrolase
GHMP kinase: Galacto-kinase, homoserine-kinase, mevalonate-kinase, phosphomevalonate-kinase
NCBI: National Center for Biotechnology Information
