# Supplementary material for: Genomic discovery of the hypsin gene and biosynthetic pathways for terpenoids in Hypsizygus marmoreus
Source: BMC Genomics. 2018 Nov 1;19:789. doi: 10.1186/s12864-018-5159-y (PMC6211417; doi:10.1186/s12864-018-5159-y)
Supplement: Supplementary file 1 — Figure S1. K-mer frequency of genomic reads. Figure S2. Sequence coverage histogram. Figure S3. Read coverage and GC content plot. Figure S4. Genome sizes and gene numbers of Agaricomycetes. Figure S5. Terpene synthase genes of 70 Agraicomycetes. Figure S6. The gene tree of terpene synthase genes of ten Agraicomycetes. Figure S7. Arrangement of conserved neighboring genes surrounding terpene synthase among Agaricomycetes (clade 3). Figure S8. Arrangement of conserved neighboring genes surrounding terpene synthase among Agaricomycetes (clade 4). Figure S9. Arrangement of conserved neighboring genes surrounding terpene synthase among Agaricomycetes (clade 5). Figure S10. Transcriptional expression of terpene synthase genes and their neighboring genes. (PDF 2772 kb) [file 12864_2018_5159_MOESM1_ESM.pdf]

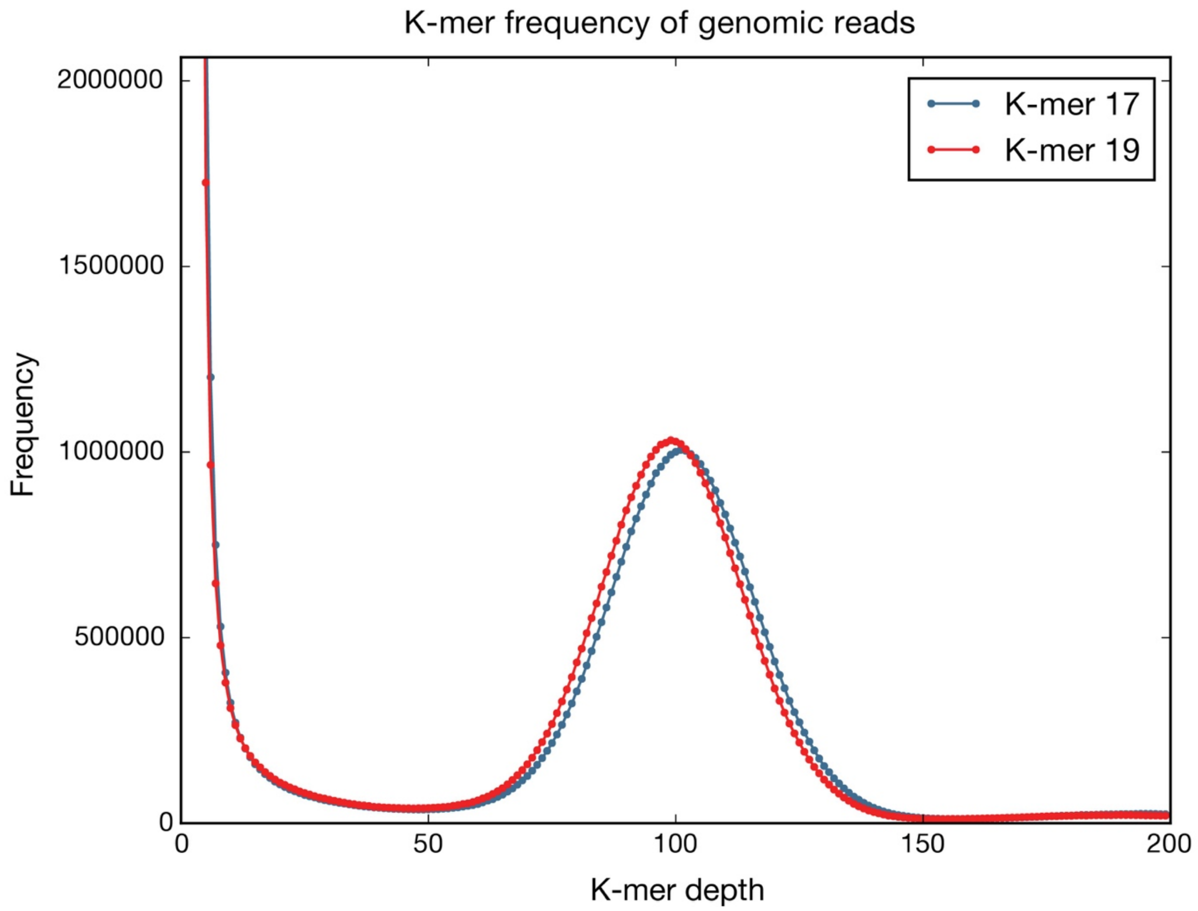

Figure S1. K-mer frequency of genomic reads. Illumina paired-end reads were used to count k-mers.

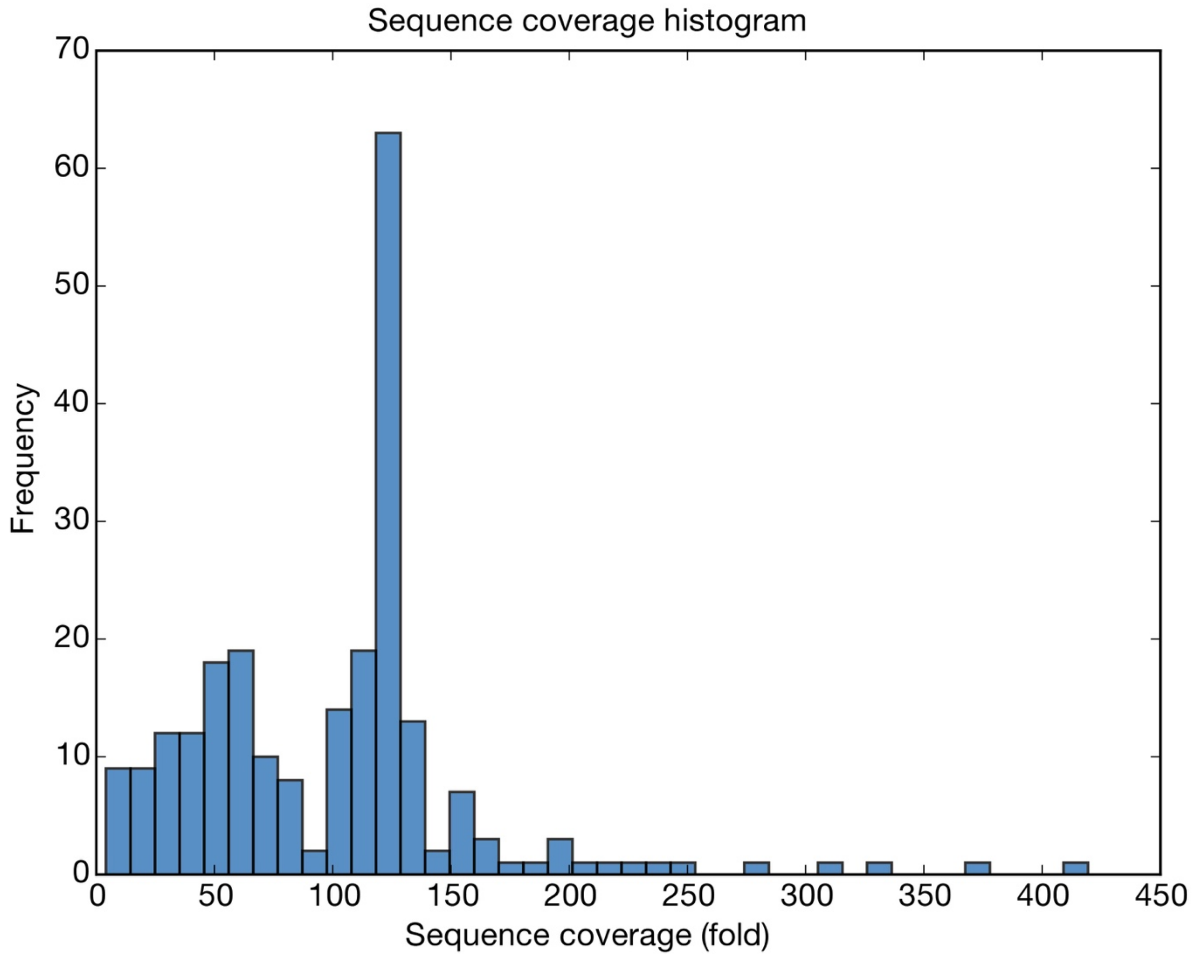

Figure S2. Sequence coverage histogram. Illumina paired-end reads were mapped into the final assembly and the sequence coverages were calculated for each scaffold using Blobology (https://github.com/blaxterlab/blobology).

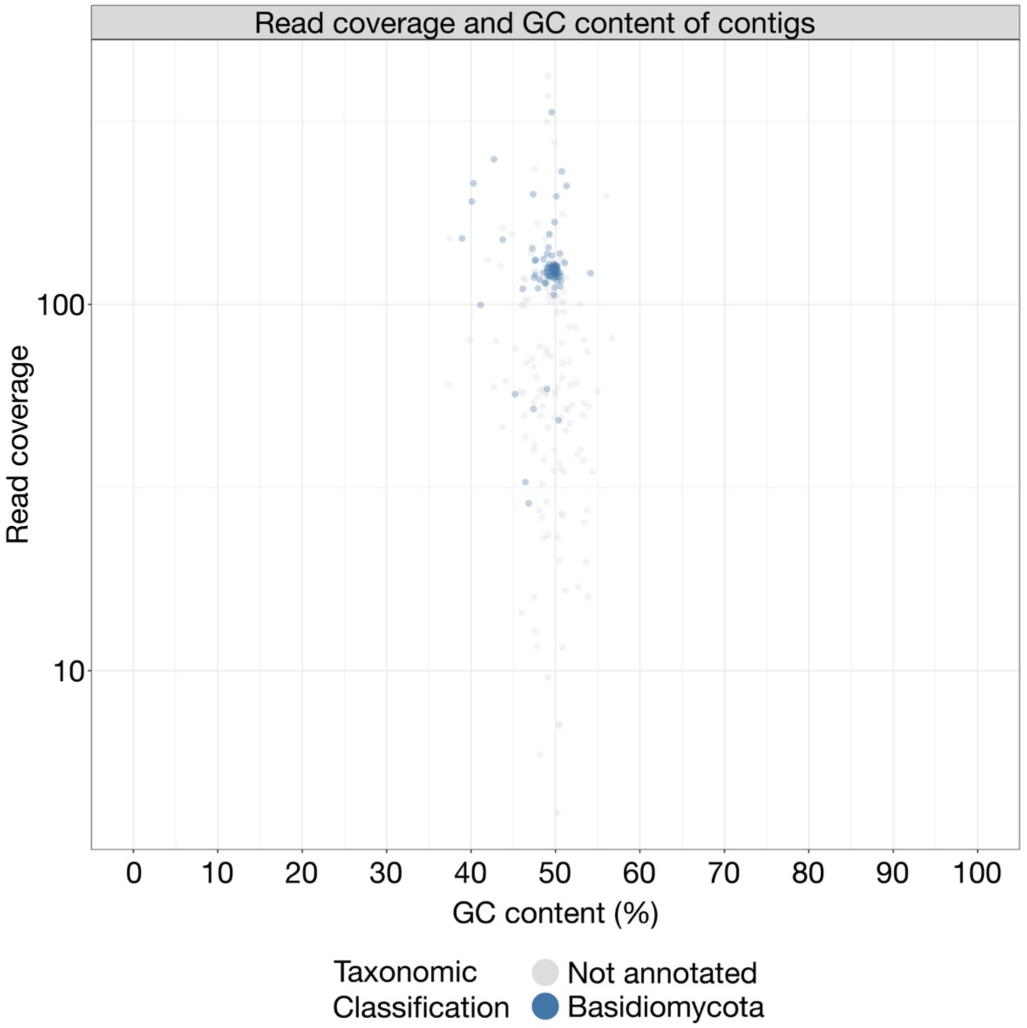

Figure S3. Read coverage and GC content plot. Illumina paired-end reads were mapped into the final assembly and the read coverages were calculated for each scaffold using Blobology (https://github.com/blaxterlab/blobology). Taxonomic classification is based on Megablast alignments to the NCBI *nt* database (ftp://ftp.ncbi.nlm.nih.gov/blast/db/).

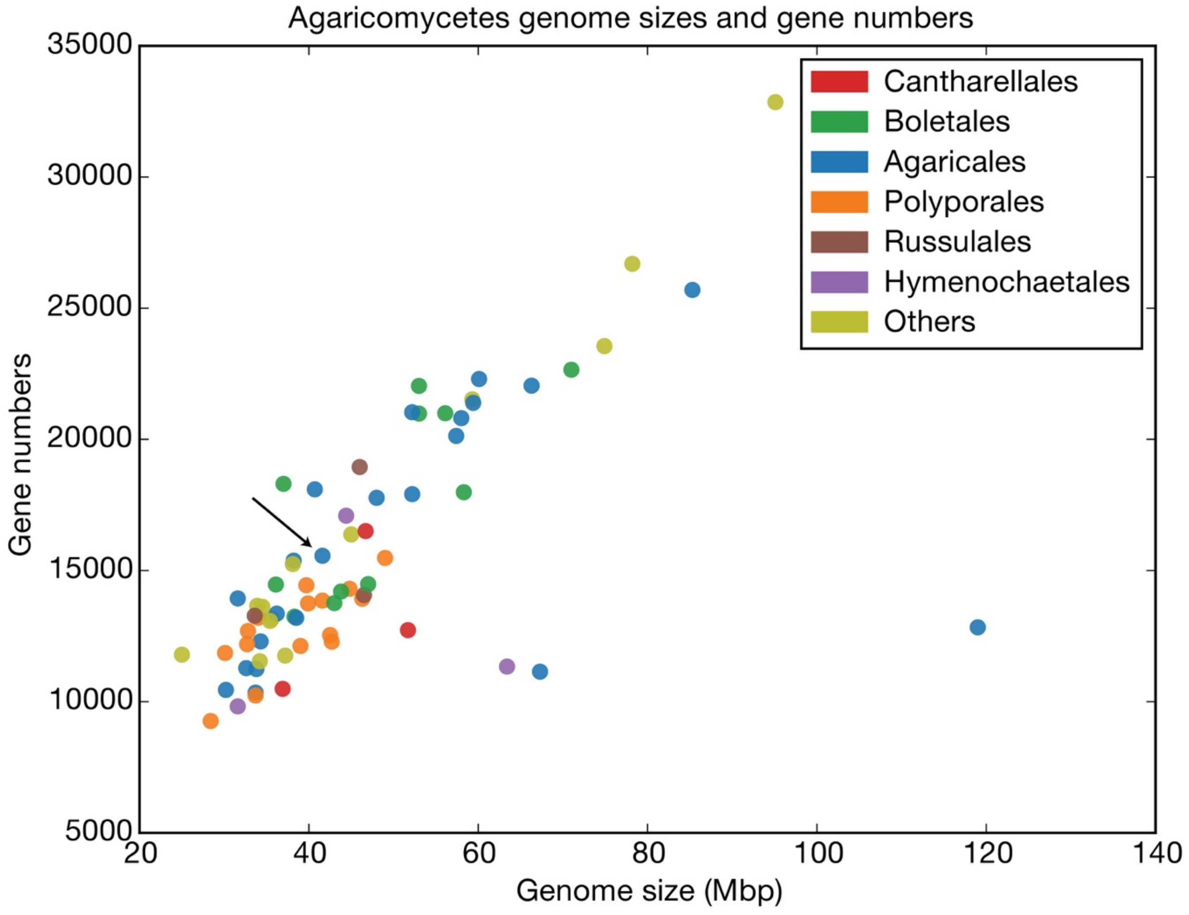

Figure S4. Genome sizes and gene numbers of Agaricomycetes. *Hypsizygus marmoreus* is indicated by the arrow.

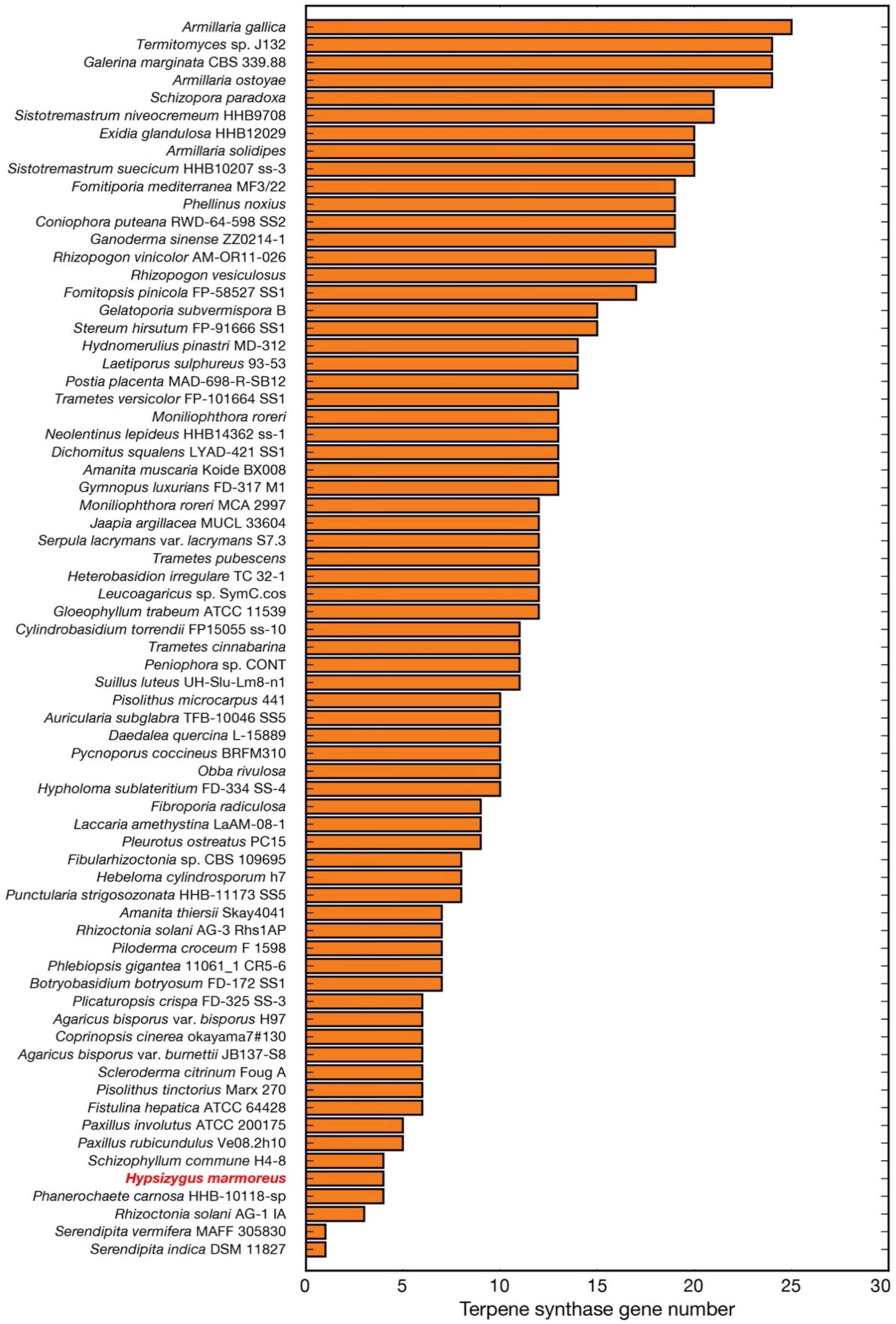

Figure S5. Terpene synthase genes of 70 Agraicomycetes. Terpene synthase genes were predicted by ortholog searches of known terpene synthase genes from *Coprinus cinereus*.

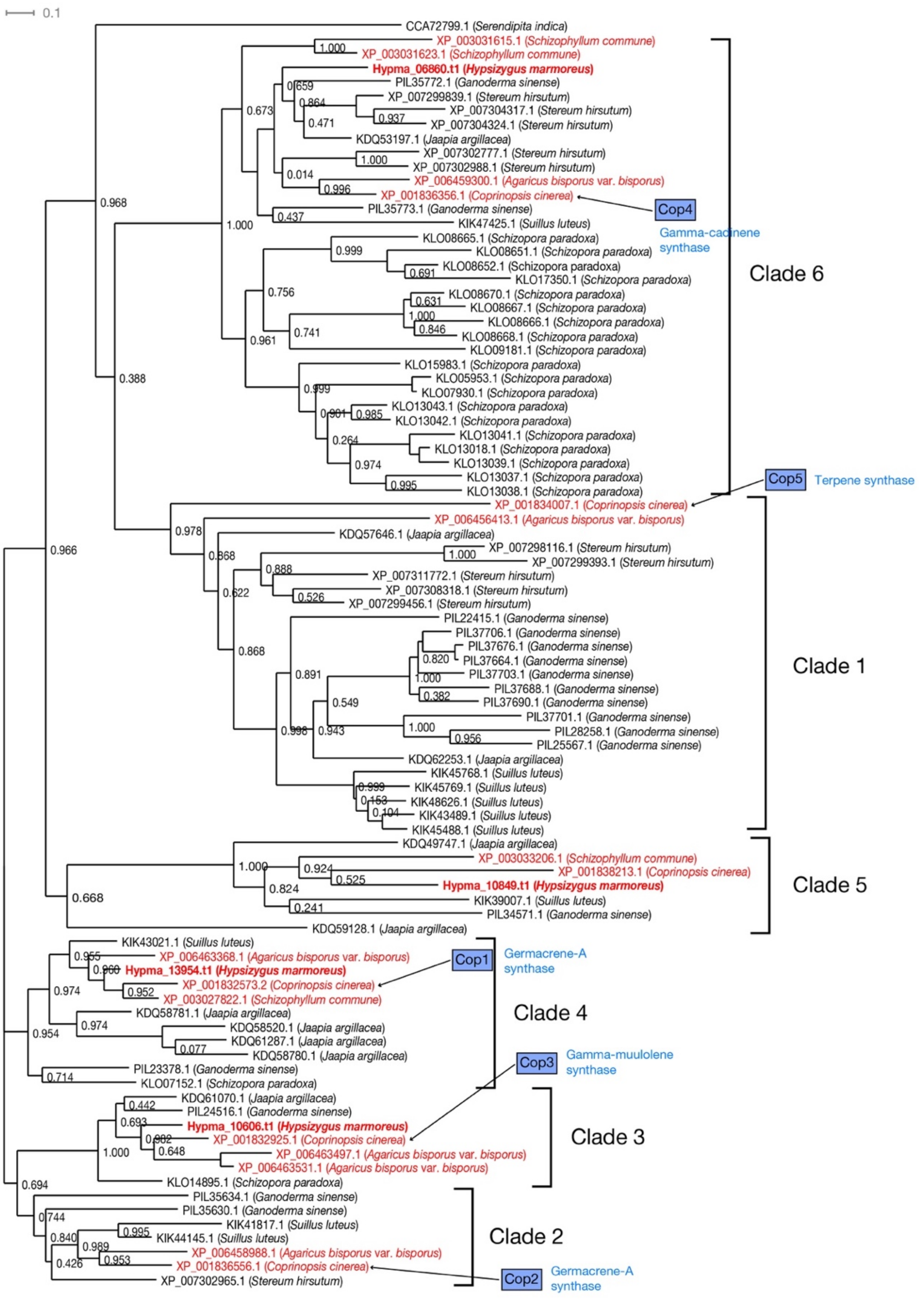

Figure S6. The gene tree of terpene synthase genes of ten Agraicomycetes. Terpene synthase genes were predicted by ortholog searches for known *Coprinus cinereus* genes. Mafft 7.273 and FastTree 2.1.3 were used for sequence alignment and tree building, respectively. Clade classification was consistent with the gene tree in Figure 6. Agaricales genomes are marked with red color.

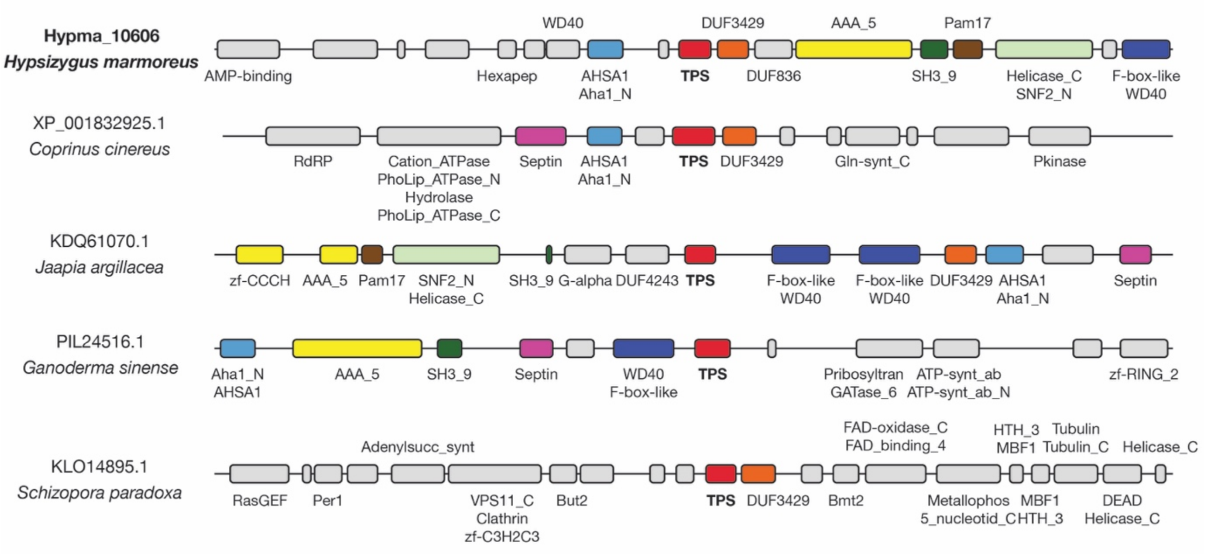

Figure S7. Arrangement of conserved neighboring genes surrounding terpene synthase among Agaricomycetes (clade 3). It displays 40-kbp regions encompassing terpene synthases where each gene is labeled with Pfam annotation when available. Orthologous genes are marked with the same color.

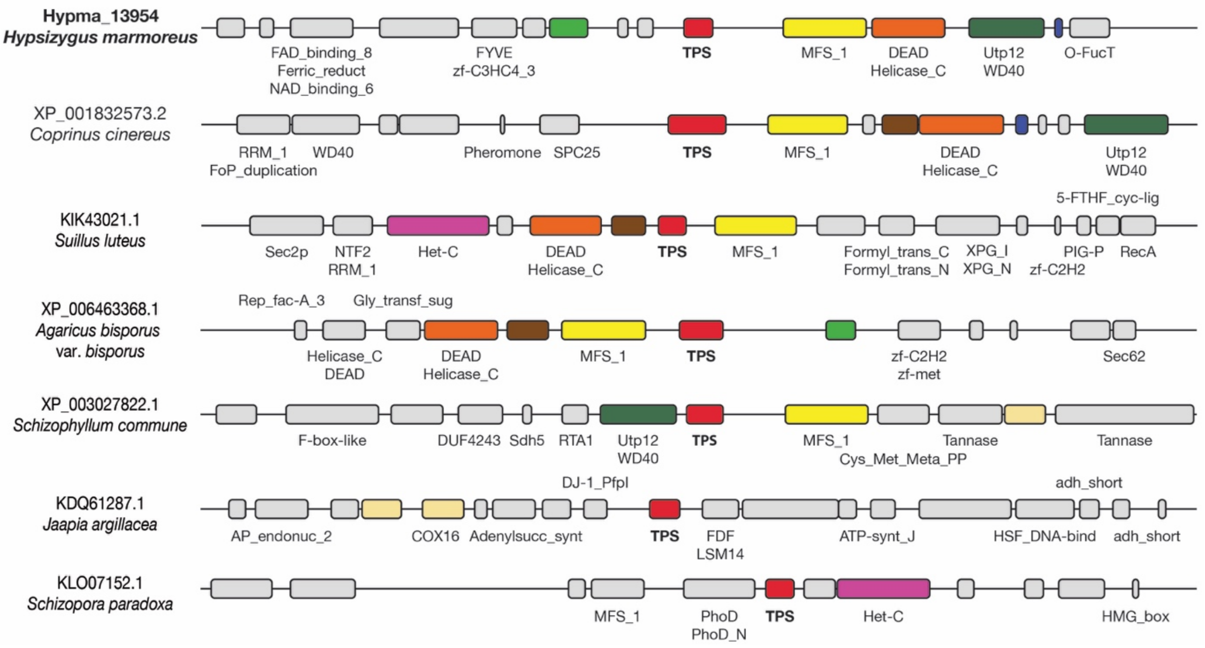

Figure S8. Arrangement of conserved neighboring genes surrounding terpene synthase among Agaricomycetes (clade 4). It displays 40-kbp regions encompassing terpene synthases where each gene is labeled with Pfam annotation when available. Orthologous genes are marked with the same color.

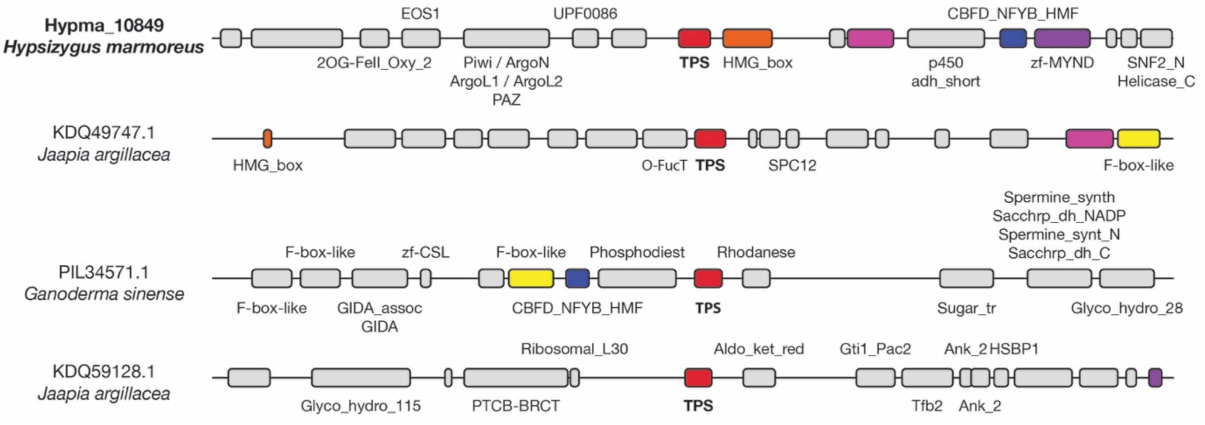

Figure S9. Arrangement of conserved neighboring genes surrounding terpene synthase among Agaricomycetes (clade 5). It displays 40-kbp regions encompassing terpene synthases where each gene is labeled with Pfam annotation when available. Orthologous genes are marked with the same color.

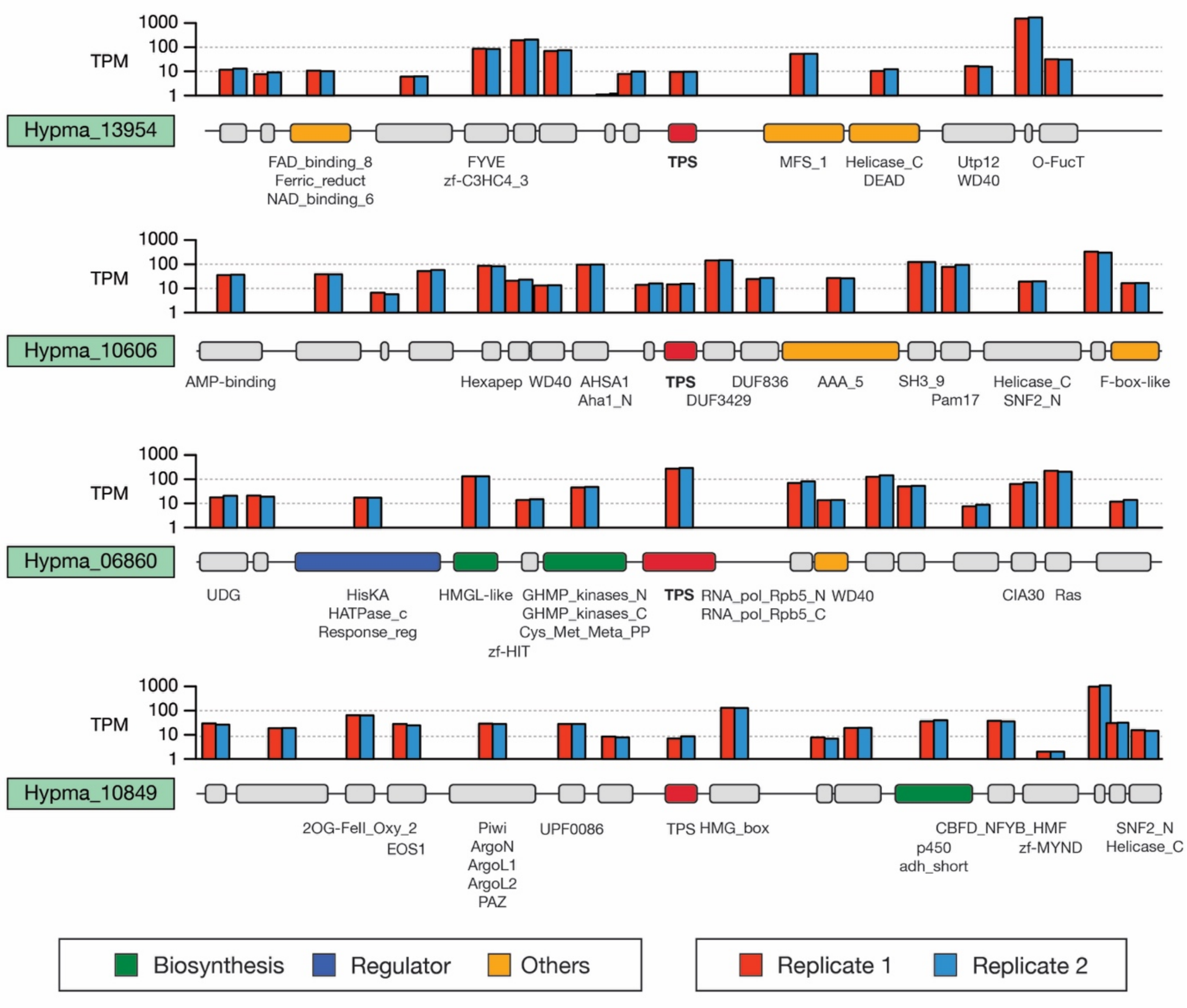

Figure S10. Transcriptional expression of terpene synthase genes and their neighboring genes. The mRNA molecules were extracted from hyphae and two duplicated sequencing libraries were generated and sequenced. Hisat 2.0.2 was used for read alignment and HTSeq 0.10.0 was used for aligned reads count for each gene. Transcripts per million (TPM) values were calculated for normalization. Secondary metabolism genes are marked by colors based on smCOG annotations.
